# Fluid Field Modulation in Mass Transfer for Efficient Photocatalysis

**DOI:** 10.1002/advs.202203057

**Published:** 2022-08-11

**Authors:** Baoying Dai, Yihao Zhou, Xiao Xiao, Yukai Chen, Jiahao Guo, Chenchen Gao, Yannan Xie, Jun Chen

**Affiliations:** ^1^ State Key Laboratory of Organic Electronics and Information Displays & Institute of Advanced Materials (IAM) Jiangsu Key Laboratory for Biosensors Jiangsu National Synergetic Innovation Center for Advanced Materials (SICAM) Nanjing University of Posts and Telecommunications Nanjing 210023 China; ^2^ Department of Bioengineering University of California, Los Angeles Los Angeles CA 90095 USA; ^3^ State Key Laboratory of Materials‐Oriented Chemical Engineering College of Materials Science and Engineering Nanjing Tech University Nanjing 210009 China

**Keywords:** artificial cilia, fluid field, mass transfer, micro/nanomotors, photocatalysis

## Abstract

Mass transfer is an essential factor determining photocatalytic performance, which can be modulated by fluid field via manipulating the kinetic characteristics of photocatalysts and photocatalytic intermediates. Past decades have witnessed the efforts and achievements made in manipulating mass transfer based on photocatalyst structure and composition design, and thus, a critical survey that scrutinizes the recent progress in this topic is urgently necessitated. This review examines the basic principles of how mass transfer behavior impacts photocatalytic activity accompanying with the discussion on theoretical simulation calculation including fluid flow speed and pattern. Meanwhile, newly emerged viable photocatalytic micro/nanomotors with self‐thermophoresis, self‐diffusiophoresis, and bubble‐propulsion mechanisms as well as magnet‐actuated photocatalytic artificial cilia for facilitating mass transfer will be covered. Furthermore, their applications in photocatalytic hydrogen evolution, carbon dioxide reduction, organic pollution degradation, bacteria disinfection and so forth are scrutinized. Finally, a brief summary and future outlook are presented, providing a viable guideline to those working in photocatalysis, mass transfer, and other related fields.

## Graphical abstract

Mass transfer behavior of reactive species generated during photocatalysis plays a vital role in determining photocatalytic performance, which can be modulated by fluid field parameters. In this review, typical light‐/bubble‐propelled micro/nanomotors and magnet‐actuated artificial cilia with tailored structure and ingenious composition developed for efficient fluid field‐manipulated mass transfer as well as their applications in photocatalysis are scrutinized.

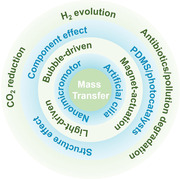

## 1 Introduction

The current energy shortage and environmental crisis throughout the world pose an imperious need for developing renewable energy sources with the characteristics of zero carbon emission, high energy conversion efficiency, and high energy density.^[^ ^1^, ^2^, ^3^, ^4^, ^5^, ^6^, ^7^, ^8^, ^9^, ^10^, ^11^, ^12^, ^13^, ^14^, ^15^ ^]^ In this context, more and more focuses were allocated to viable and robust technologies for generating green energy and thus substituting traditional fossil fuels.^[^ ^16^, ^17^, ^18^, ^19^, ^20^, ^21^, ^22^, ^23^, ^24^, ^25^, ^26^, ^27^, ^28^, ^29^, ^30^, ^31^, ^32^ ^]^ For example, piezoelectric/triboelectric/pyroelectric nanogenerators, especially the recently invented magnetoelastic generators,^[^ ^22^, ^24^, ^25^, ^26^ ^]^ can scavenge surrounding renewable energy (such as human walking and running, heart beating, natural water flow, wind, environmental temperature variation, and so forth) to produce electricity for power supply or healthca,^[^ ^33^, ^34^, ^35^, ^36^, ^37^, ^38^, ^39^, ^40^, ^41^, ^42^, ^43^, ^44^, ^45^, ^46^, ^47^, ^48^, ^49^, ^50^, ^51^, ^52^ ^]^ electrocatalytic technology can undergo oxygen reduction and oxygen/hydrogen evolution reactions under bias voltage to yield clean energy,^[^ ^53^, ^54^, ^55^, ^56^, ^57^, ^58^, ^59^, ^60^ ^]^ and environment‐friendly batteries are developed and applied in electrochemical energy storage and conversions.^[^ ^61^, ^62^, ^63^, ^64^, ^65^ ^]^ Notably, among these viable technologies, photocatalytic technology can convert inexhaustible solar energy into chemical energy, e.g., splitting water into green fuel hydrogen, reducing carbon dioxide into carbon oxide or methane, fixing nitrogen, degrading organic pollutions and antibiotics, and disinfecting bacteria and viruses, which shows greater potential in alleviating the global energy and environmental issues.^[^ ^66^, ^67^, ^68^, ^69^, ^70^, ^71^, ^72^, ^73^ ^]^ In the past decades, numerous efforts have been devoted to elevate photocatalytic efficiency from the perspective of broadening light absorption (in terms of developing narrow‐band gap photocatalysts, constructing upconversion‐photocatalytic composites, depositing noble metal nanoparticles with surface plasmonic resonance effect, and so forth),^[^ ^74^, ^75^, ^76^, ^77^, ^78^ ^]^ promoting photoexcited carrier migration and separation (in terms of constructing Z‐scheme, S‐scheme, homogeneous/heterojunction photocatalysts, introducing built‐in electric field, depositing co‐catalysts),^[^ ^79^, ^80^, ^81^, ^82^, ^83^, ^84^, ^85^, ^86^ ^]^ as well as boosting surface molecule activation.^[^ ^87^ ^–^ ^91^ ^]^ Although great achievements have been witnessed, solar energy conversion efficiency in photocatalysis is still too low to meet the demand for industrialization. As it is well known, photocatalytic reaction is a very fast process, and the photoinduced oxidants and reductants (such as hydroxyl radicals and super oxide radicals) are short‐live species that are only available near the photocatalyst surface or interface.^[^ ^92^ ^–^ ^94^ ^]^ Hence, besides the above‐mentioned strategies, the efficient transportation of active species (including photoexcited electrons, holes, hydroxyl radicals, superoxide radicals) to photocatalyst surface or interface is also a decisive factor for achieving high‐efficient photocatalytic performance, which needs to be taken into account when designing photocatalytic systems.^[^ ^95^ ^–^ ^98^ ^]^

Profited from the rapid development of theory simulation calculation and nanomaterials science and technology, we got deep insight into the kinetic process and character of photocatalytic physical and chemical reactions as well as the seminal role of mass transfer behavior in photocatalysis.^[^ ^99^ ^–^ ^102^ ^]^ Specifically, mass transfer behavior has a crucial impact on the adsorption and desorption characters of reactive species (e.g., water, organic dye and CO_2_ molecules) on photocatalyst surface which determine the utilization efficiency of active species (promoting photoexcited electrons and holes to take part in photocatalytic redox reaction before recombination) and the release of photocatalyst active sites (for achieving sustainable photocatalysis), respectively, and thus modulate final photocatalytic performance.^[^ ^103^ ^–^ ^113^ ^]^ Based on this, some judicious fluid field‐facilitated mass transfer manipulation methods based on photocatalyst composition and structure design were developed and applied to boost photocatalytic efficiency in recent years.^[^ ^114^ ^–^ ^117^ ^]^ Notably, different kinds of self‐propelled micro/nanomotor photocatalysts with Janus structures and multicompositions were constructed for facilitating mass transfer kinetics (via self‐thermophoresis, self‐diffusiophoresis and bubble‐propulsion mechanisms) and thus boosting photocatalytic performance (including organic pollution degradation rate, hydrogen evolution property, carbon dioxide reduction activity, and disinfection efficiency).^[^ ^118^ ^–^ ^122^ ^]^ In addition, inspired by natural cilia structure, magnet‐actuated flexible artificial cilia photocatalysts were also developed, showing improved mass transfer kinetics and enhanced photocatalytic performance as well.^[^ ^123^ ^–^ ^125^ ^]^ These impactful studies conducted in the past decades gave access to great progresses and achievements in the investigation on mass transfer kinetics during photocatalysis, providing robust strategies for improving photocatalytic performance, which have not been systematically examined yet as far as we know.^[^ ^126^ ^]^ Therefore, a critical survey centering on fluid field‐modulated mass transfer for high‐efficient photocatalysis is of great importance to present an available guideline for the researchers who work in this field and other related disciplines.

**Figure 1:**
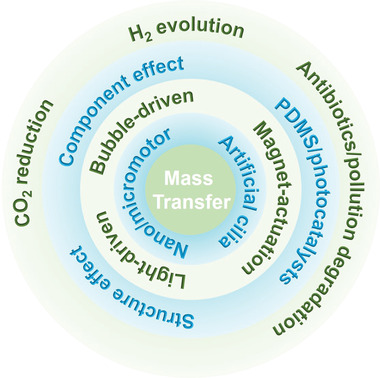
Diagram of the recent progress and applications of mass transfer‐promoted photocatalytic performance.

Hence, in this review, we will detail the mechanism how mass transfer manipulates photocatalytic reaction process and the progress in theoretical simulation calculations based on computational fluid dynamics (CFD) for optimizing mass transfer parameters. Thereafter, we will examine representative photocatalytic micro/nanomotors and bioinspired artificial cilia‐based photocatalysts with tailored structure and composition developed for promoting mass transfer via specific self‐propulsion mechanism and thus facilitating photocatalytic performance, as depicted in **Figure** **1**. Finally, we will provide a summary along with future outlook from the points of material composition, structure and function design, experimental characterization and theoretical simulation calculation on mass transfer behavior, as well as their practical applications. This review is expected to depict a roadmap that directs and drives the future development of mass transfer manipulation, photocatalytic micro/nanomotors, artificial cilia, and other related fields.

## 2 Mechanism of Mass Transfer Impacting Photocatalysis and Simulation Calculations

### 2.1 Mass Transfer Property in Photocatalysis

**Figure 2:**
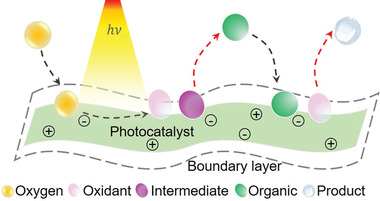
Schematic diagram of mass transfer influencing photocatalytic efficiency. Under light irradiation, some of photoexcited electrons and holes move to the surface of photocatalyst for photocatalytic redox reactions. Oxygen dissolved in solution and target organics adsorb on the surface of photocatalyst to react with active species (i.e., electrons, holes, etc.) and form oxidation production and intermediate, respectively, which may adsorb on or desorb from photocatalytic surface, forming final redox reaction production. Black and red dashed lines represent adsorption and desorption routes of the above‐mentioned species, respectively.

During a photocatalytic process, photogenerated electrons get excited from the valence band of semiconductor photocatalyst and move to the conduction band under light illumination, leaving the same number of photogenerated holes at the valence band. Thereafter, a part of photogenerated electrons and holes move to photocatalyst surface and take part in photocatalytic reduction and oxidation reactions, respectively. In a typical photocatalytic redox reaction, mass transport behavior of active species and target reactants can be mainly divided into four cases (**Figure** **2**).^[^ ^127^ ^]^ In the first case, oxygen molecule dissolved in the reactive solution and water molecule may adsorb onto photocatalyst surface and react with photoexcited electrons and holes to generate superoxide radical and hydroxyl radical. In the second case, target organics adsorb on the surface of photocatalyst to react with active species. In the third case, oxidation products such as intermediates and final products originated from photocatalytic redox reactions in case two desorb from surface to bulk reactive solution. In the fourth case, photoinduced reactive oxidative and reductive species desorb from photocatalyst surface and form final products. Therefore, the above‐mentioned mass transfer behavior has a decisive impact on final photocatalytic property, which was always ignored during photocatalytic reactions unfortunately. One possible reason might be that mass transfer information is difficult to describe, especially for bulk photocatalytic containers due to the chaotic fluid state of photocatalytic system. In this regard, photocatalytic microreactor with the advantages of uniform irradiation, stable laminar flow and shorter molecular diffusion distances was selected as a platform to study photocatalytic kinetics, in which the aforementioned four mass transport processes can be divided into two categories (internal and external mass transport processes) based on the places they exist.^[^ ^128^ ^–^ ^130^ ^]^ It is reported that external mass transfer rate (*r*_external_) can be described with Equation (1) on the basis of two‐film model^[^ ^127^ ^]^

(1) $$r_{\mathrm{external}}=k_{\mathrm{external}}\;\left(c_{\mathrm{bulk}}-c_{\mathrm{surface}}\right)$$

where *k*_external_, *c*_bulk_, and *c*_surface_ are the external mass transfer coefficient, bulk solution concentration and catalyst surface concentration, respectively. Additionally, *k*_external_ can be experimentally determined for an immobilized photoreactor, which is related to Reynolds number (*Re*) as expressed in Equations (2) and (3)^[^ ^131^ ^]^

(2) $$k_{\mathrm{external}}\;\left({m\;s}^{-1}\right)=3.49\times 10^{-7}\times {Re}^{0.77}$$

(3) Re=ρνd/μ

in which *ρ*, *v*, *d*, and *µ* represent the fluid density, flow rate, diameter of photocatalytic reactor, and viscosity coefficient, respectively, and *Re* decides the flow pattern of photocatalytic reaction solution. Therefore, it can be concluded that external mass transfer efficiency is positively correlated to the flow rate of reactive solution, and a well‐stirred system possesses high external mass transfer efficiency, resulting in high photocatalytic activity.

As for internal mass transfer behavior, it is predominately dependent on the porosity of photocatalyst because internal mass transfer process includes various parameters (e.g., pore dimensions and molecular diffusion coefficient), which is difficult to calculate and describe exactly. In this context, an indirect approach was proposed by Weisz and Prater via surface reaction rate using the Weisz‐Prater modulus (*φ*′), which can be depicted as Equation (4)^[^ ^132^ ^]^

(4) $$\varphi^{\prime }=\frac{r^{\prime }d_{L}^{2}\tau }{D\varepsilon C^{*}}$$

where *r*′, d_L_, *τ*, *D*, *ɛ*, and C* stand for the reaction rate per catalyst unit, characteristic dimension of the deposit, tortuosity of catalyst, diffusion coefficient, catalyst porosity, and the concentration at interface, respectively. When *φ*′ is higher than 1, internal mass transfer property should be considered for optimizing photocatalytic performance. Thus, both the internal and external mass transfer rates play a crucial role in modulating photocatalytic redox reaction processes, especially in immobilized photocatalyst systems. Moreover, photocatalytic redox reactions take place at photocatalyst surface, and the capture of photogenerated electrons and holes undergoes in the presence of oxygen dissolved in photocatalytic system and water molecules. Therefore, the adsorption process of these target species on photocatalyst surface is also a vital factor determining final photocatalytic activity, which is impacted by the parameters of photocatalytic reactive solution fluid field.

### 2.2 Theoretical Calculation

**Figure 3:**
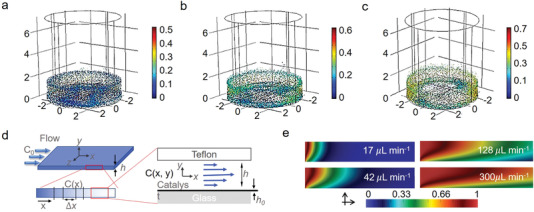
Catalyst particle speed and trajectories for different stirring speeds: a) 100, b) 300, and c) 475 rpm. Reproduced with permission.^[^ ^137^ ^]^ Copyright 2019, American Chemical Society. d) Schematic diagram of the flow domain geometry (out of scale) and coordinate system, a simplified unidirectional view of the reaction zone with average reagent concentration in the gap, and details of the flow field in the reaction cell. e) Numerical simulations of the concentration field inside the reaction cell for different flow rates. Reproduced with permission.^[^ ^138^ ^]^ Copyright 2018, Elsevier.

Based on the above discussions, it can be found that the mass transfer behavior of photocatalysts or active species are greatly dependent on the characteristics of fluid field, especially for photocatalyst films and immovable photocatalyst substances. Hence, for achieving excellent photocatalytic property, it is highly desirable to optimize kinetic characteristics of the reactant molecules, species and photocatalysts. Theoretical simulation calculation based on CFD presents a promising method to design and scale‐up photocatalytic reaction in practical applications.^[^ ^133^ ^–^ ^136^ ^]^ For example, Yesid imitated the speed and trajectories of catalyst particles under different stirring speeds, and the results suggested that average particle speed increased with the increases of stirring speed (**Figure** **3**a–c), demonstrating the significant role of high fluid flow speed in facilitating mass transfer during photocatalysis.^[^ ^137^ ^]^ Additionally, on the grounds of radial stirring system used in their model, there was always a zero‐velocity zone at the center of the reactor's geometry. It implied that a bulk reactor was not a wise choice to achieve high photocatalytic efficiency. Based on this, Satuf et al. assessed the intrinsic kinetic constants in photocatalytic reactors using a planer microreactor (Figure 3d).^[^ ^138^ ^]^ The results indicated that under the same irradiation condition, faster flow speed led to lower degradation percent due to the lower residence time of solution in the microreactor. Moreover, compared to bulk reactor, photocatalytic reaction in microreactors was more stable because of the disturbance of chaotic fluid. Once the first drop of solution flowed out from the microreactor, the concentration status inside the reactor would stay the same. As depicted in Figure 3e, the concentration distribution under different flow rates was described by numerical simulations, and the target solution was almost fully degraded under a lower flow rate of 17 µL min^–1^, making the rear part of the microreactor redundant.^[^ ^138^ ^]^ On the opposite, the target solution was partly degraded under higher flow rates (42, 128, and 300 µL min^–1^). These works implied that flow rate was a two‐edged sword for modulating mass transfer behavior and corresponding photocatalytic property, demonstrating the importance of kinetic constant optimization over photocatalytic system for achieving high‐efficient photocatalysis.

**Figure 4:**
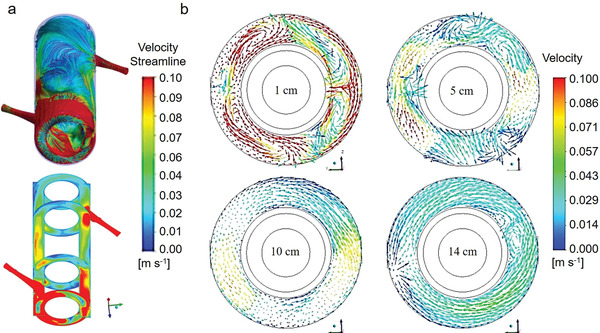
a) Streamlines and contours of velocity magnitude of the reactor and (b) velocity vectors along the axial plane at different distances from the bottom of the reactor. Reproduced with permission.^[^ ^140^ ^]^ Copyright 2016, Elsevier.

Except the above‐mentioned flow speed of photocatalytic reactive fluid field, flow pattern also has a vital influence on mass transfer as well as photocatalytic performance. For example, Pareek and co‐authors investigated the fluid field flow pattern in an annular slurry reactor and various flow patterns were detected in different parts of the reactor.^[^ ^139^ ^]^ Specifically, flow pattern was in chaos and a vortex can be seen near the inlet of the reactor, whereas the flow pattern was uniform and no disturbance was observed in the tube. It disclosed that the modulation on flow pattern was of great importance to optimize mass transfer property and thus maximize the efficiency of photocatalytic system. In addition, Casado and co‐authors systematically calculated the intrinsic kinetic parameters of photocatalytic process by CFD model and a similar conclusion was attained (**Figure** **4**).^[^ ^140^ ^]^ These simulation calculation results demonstrated that mass transfer flow was not uniform through the fluid medium, unraveling that the shorter photocatalytic reactor in length was more beneficial for increasing mass transfer efficiency during photocatalytic reactions.

Previous theory simulation studies verified that the mass transfer behavior during photocatalytic reactions can be modulated by fluid flow parameters, including both fluid pattern and flow speed. In general, turbulent flow pattern and relative higher flow speed would give rise to higher mass transfer rate, and thus result in boosted photocatalytic property. However, too fast mass transfer process may have a negative impact on the efficiency of photocatalytic reactors due to the lower residence time of reactive species on the surface of photocatalysts. In addition, Lammertink and co‐workers investigated the limitation of mass transfer in photocatalytic performance via photodegrading Bisphenol A over TiO_2_ microreactor and numerical simulation calculation, and the results showed that the impact of mass transport on photocatalytic property was limited under high light intensity (photon fluxes above 25 mW cm^–2^) and this mass transport limitation would result in an undervaluation of photocatalytic surface reaction rate constant.^[^ ^141^ ^]^ It should be pointed out that the exponents of reaction rate with respect to light intensity were about 0.25 and 1 without and with the consideration of mass transfer limitation factor, respectively, which further stressed the importance of a correct inclusion of mass transfer limitation when designing photocatalytic reaction system in practical applications. From this perspective, it is necessary to optimize kinetic properties of mass transfer and avoid the negative effect of mass transfer limitation on photocatalysis for achieving high‐performance photocatalytic system.

## 3 Design of Mass‐Transfer Boosted Photocatalytic Systems

Based on the investigations on mass transfer process via theoretical simulation calculation, more and more photocatalysts with ingenious structures and subtle functions were developed to facilitate photocatalytic activity via manipulating kinetic process of mass transfer.^[^ ^142^ ^–^ ^144^ ^]^ Among them, photocatalyst‐based micro/nanomotors and cilia‐like photocatalyst films with viable structure design have aroused intensive attentions, exhibiting great potential in promoting mass transfer and thus enhancing photocatalytic performance, which will be summarized and discussed below.^[^ ^145^, ^146^ ^]^

### 3.1 Micro/Nanomotor‐Imparted Mass Transfer

Self‐propelled micro/nanomotors are widely presented in nature, such as motile cells and viruses, which provided a viable strategy to realize self‐transport and controllable crystallization, showing promising prospect of practical applications in environmental remediation, target drug delivery, active assembly, and so forth.^[^ ^147^ ^–^ ^155^ ^]^ Inspired by nature, researchers explored, and designed numerous self‐driven motors based on different propulsion mechanisms, such as self‐thermophoresis and self‐diffusiophoresis.^[^ ^156^ ^–^ ^159^ ^]^ These constructed micro/nanomotors seem to have certain superpower to convert light and chemical energy into their own kinetic energy for specific purpose.^[^ ^160^ ^–^ ^164^ ^]^ Importantly, with the fast development of photocatalytic technology, many photoactive materials have been employed to fabricate light‐driven and water/fuel‐driven micro/nanomachines.^[^ ^142^, ^165^ ^]^ It is beneficial for the improvement of photocatalytic property in return due to the vital ability of photocatalyst‐based micro/nanomotors to communicate with the surrounding particles, accelerating mass transfer during photocatalytic reactions. By now, many self‐propelled photocatalyst motors (for instance, light‐driven and bubble‐driven motors) have been designed and introduced into photocatalytic system for enhancing photocatalytic activity, and some typical representatives were summarized in **Table** **1**.

**Table 1:** Summary of some typical micro/nanomotors for enhancing photocatalytic property via modulating mass transfer behavior

Motor type	Structure design	Mechanism	Catalytic applications	Ref.
Light‐driven	CB/g‐C_3_N_4_	Self‐thermophoresis	H_2_ evolution, enhanced by 2.1 times	[169]
	CB/P25		H_2_ evolution, boosted by 2.3 times	[169]
	TiO_2_‐Au Janus	Self‐diffusiophoresis	Methyl blue degradation, increased by 48% (first‐order rate constant)	[174]
	Au‐WO_3_@C Janus		Sodium‐2,6‐dichloroindophenol degradation, boosted by 70%	[177]
	TiO_2_‐Fe		Rhodamine 6G degradation, improved by 40%	[179]
	Star‐shaped BiVO_4_		Yeast cell disinfection, increased by 40%	[181]
	CNT/TiO_2_		Phenol conversion, enhanced by 37%	[180]
	ZnO/Pt Janus		Picric acid and methyl blue degradation, boosted by 1.2 and 1.9 times, respectively	[176]
Bubble‐driven	TiO_2_/Au/Mg Janus	Mg reacts with water	Bacillus globigii spore disinfection, increased by 75%	[186]
	Pt/TiO_2_/Au semishell	Pt reacts with H_2_O_2_	Methyl blue degradation, improved by 70%	[187]
	Pt/TiO_2_/Pt nanotube	Pt reacts with H_2_O_2_	Rhodamine B decomposition, boosted by 30%	[188]
	Yolk–shell Au‐CdS	CdS reacts with H_2_O	H_2_ evolution, increased by about 8.4 times	[190]

#### 3.1.1 Light‐Driven Photocatalyst‐Based Micro/Nanomotors

##### Self‐Thermophoresis Mechanism

**Figure 5:**
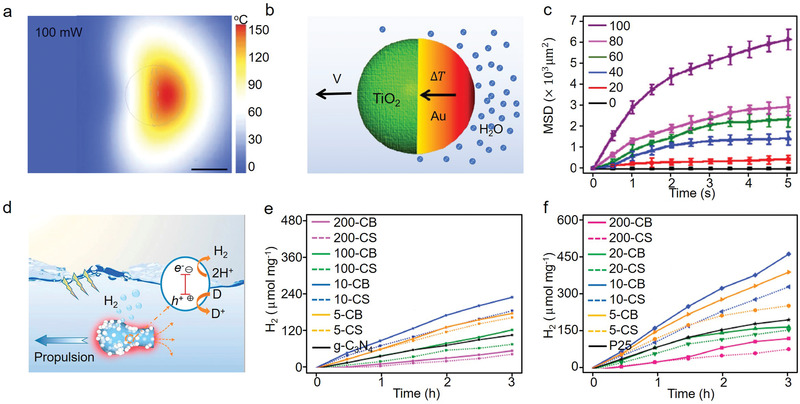
a) Theoretical simulation of the photothermal effect of a Janus micromotor under laser irradiation with a power of 100 mW cm^−2^. b) Schematic mechanism of near‐infrared‐light‐propulsion of Au‐TiO_2_ Janus motor converts light to heat, accelerating the motion of water molecules (blue spheres), and c) dynamic mean squared displacements (MSD) of Au‐TiO_2_ Janus motor with a size of 10 µm under different intensities of focused laser power. Reproduced with permission.^[^ ^168^ ^]^ Copyright 2016, Springer. d) Schematic diagram of CB motor promoting mass transfer and enhancing photocatalytic hydrogen evolution performance, and the corresponding photocatalytic hydrogen evolution activities of e) CB/g‐C_3_N_4_ and f) CB/P25 samples at room temperature under Xe lamp irradiation. Reproduced with permission.^[^ ^169^ ^]^ Copyright 2021, Elsevier.

Light‐driven micro/nanomotors are mainly constructed with photocatalyst and photothermal material (e.g., photothermal materials and carbon materials) or plasmonic material (noble materials) by forming Janus sphere particles. Under light illumination, an asymmetric heat field would be built across the Janus structure motor, which would induce temperature difference and lead to fluid flow via self‐thermophoresis.^[^ ^166^, ^167^ ^]^ In this context, He's group reported a near‐infrared‐light‐driven Janus microcapsule motor Au‐TiO_2_, which exhibited a high propulsion property with the migration speed of 42 µm s^–1^ in water ascribed to self‐thermophoresis effect (**Figure** **5**a,b).^[^ ^168^ ^]^ As illustrated in Figure 5c, a higher speed of Janus motors was detected upon the increases in the intensities of near‐infrared‐light power changed from 20 to 100 mW cm^–2^ and the mean square displacement (MSD) was obviously boosted from 350 (irradiated under the light of 20 mW cm^–2^) to 6500 µm^2^ (irradiated upon the light of 100 mW cm^–2^) in 5 s. Therefore, it can be reasonable predicted that the transfer of active species could be boosted in this process and thus photocatalytic efficiency could be improved.^[^ ^121^ ^]^

Based on the above‐discussed results, Lu and co‐authors developed a self‐propelled jet carbon micromotor (carbon bottle, CB) and combined it with photocatalysts g‐C_3_N_4_ and P25 exclusively by a simple soft‐template‐based hydrothermal method, as depicted in Figure 5d.^[^ ^169^ ^]^ The results showed that the constructed CB worked with “on‐the‐fly” mode and moved very fast under infrared light illumination, which activated mass transport flow efficiently. Consequently, the photocatalytic hydrogen evolution rates of 10 wt% CB/g‐C_3_N_4_ and 10 wt% CB/P25 were 2.1 and 2.3 times higher that of pristine g‐C_3_N_4_ and P25 (Figure 5e,f) thanks to the promoted mass transfer originated from self‐propulsion of micromotors, respectively. Additionally, compared with traditional Janus motors, the prepared jet carbon micromotor possessing the advantages of simple synthesis, low price and precious metal free was much friendlier to environment. Notably, this fabricated CB motor can be triggered in pure water without additional chemicals as fuels, benefiting its practical applications in photocatalytic hydrogen production as well as environment remediation. This work provided a new strategy for the development of self‐driven high‐performance photocatalytic systems based on self‐thermophoresis mechanism, demonstrating that Janus structure was no longer a requirement for realizing self‐propelled mass transfer during photocatalysis.

##### Self‐Diffusiophoresis Mechanism

**Figure 6:**
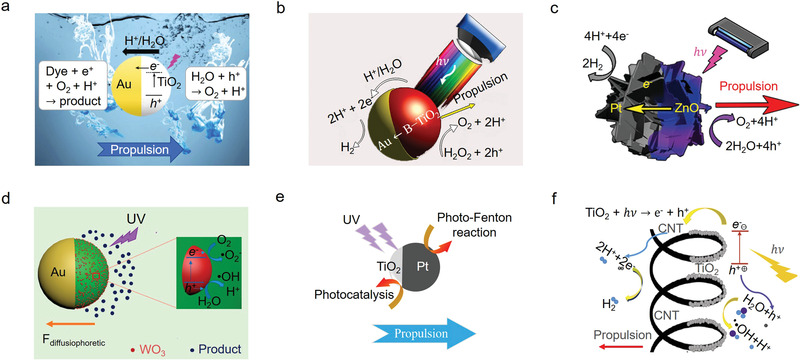
Schematic mechanisms of light‐driven micro/nanomotors. a) UV light‐induced TiO_2_‐Au Janus micromotors via self‐electrophoresis in methylal blue. Reproduced with permission.^[^ ^174^ ^]^ Copyright 2017, Springer. b) Au‐B‐TiO_2_ motor. Reproduced with permission.^[^ ^175^ ^]^ Copyright 2017, American Chemical Society. c) ZnO/Pt Janus nanomotor. Reproduced with permission.^[^ ^176^ ^]^ Copyright 2019, Wiley‐VCH. d) Light‐driven Au‐WO_3_@C. Reproduced with permission.^[^ ^177^ ^]^ Copyright 2017, American Chemical Society. e) TiO_2_‐Pt micromotors degrading rhodamine 6G by the combination of photocatalytic degradation and Fenton process. Reproduced with permission.^[^ ^179^ ^]^ Copyright 2019, Royal Society of Chemistry. f) Visible‐light‐powered CNT/TiO_2_ nanomotors. Reproduced with permission.^[^ ^180^ ^]^ Copyright 2019, Elsevier.

In addition to self‐thermophoretic mechanism for the self‐propulsion of micro/nanomotors, some light‐propelled motors were designed based on self‐diffusiophoresis originated from the osmotic pressure differences (referring to fluid motion) and diffusion potentials in the case of charges species around photocatalysts due to concentration gradient.^[^ ^170^ ^–^ ^173^ ^]^ Ren and co‐workers investigated the influence of light‐driven Janus micromotor on photocatalytic property via TiO_2_‐Au particles.^[^ ^174^ ^]^ With light irradiation, photoexcited electrons migrated to Au side from TiO_2_, and holes were left on TiO_2_ side. As a result, dye molecules were reduced by photoinduced electrons, leading to the lower dye concentration near Au side compared with that near TiO_2_ side, as depicted in **Figure** **6**a. The difference in dye concentration would cause the self‐diffusiophoretic motion of dye molecules, which led to dye solution flow and promoted itself to move continuously during photocatalytic degradation reaction. Consequently, the photocatalytic degradation first‐order rate constant was increased from 2.01 × 10^−2^ to 2.98 × 10^−2^ min^–1^.^[^ ^174^ ^]^ Similarly, Nelson and co‐authors developed multiwavelength light‐responsive Janus micromotors Au/B‐TiO_2_ in which a thin Au layer was asymmetrically deposited on the surface of black TiO_2_ microsphere with the diameter of about 3.5 µm.^[^ ^175^ ^]^ These constructed Janus micromotors could be self‐propelled under a broad wavelength range of light illumination, including ultraviolet, blue, cyan, green and red light, both in H_2_O_2_ solutions and pure H_2_O (Figure 6b). Results showed that the motion speed of micromotors decreased with the increases in wavelength and the highest speed (about 30 µm s^–1^) was achieved under entire visible light spectrum (>400 nm), corroborating the great application potentials of these constructed micromotors in photocatalysis with the utilization of full solar spectrum. Besides TiO_2_, ZnO with good photocatalytic activity was also constructed into micro/nanomotor, for example, ZnO/Pt mesoporous Janus micromotor was synthesized through a self‐aggregation method by Pumera and co‐workers, which exhibited fuel‐free light‐powered propulsion property based on interface roughness.^[^ ^176^ ^]^ Because of the different work function between Pt and ZnO, an upward band bending was formed at the interface, and electrons tended to transfer from ZnO to Pt under light irradiation, leaving holes on the valence band of ZnO to decompose pure water into protons and oxygen (Figure 6c). Consequently, the protons existed in the medium would be reduced into hydrogen at Pt side, and meanwhile, the protons would transport from ZnO to Pt ascribed to proton concentration gradient, forming a self‐diffusiophoretic flow for the propulsion of micromotor and an electric dipole for asymmetric ZnO/Pt particle, which led to a diffusiophoretic motion and increased fuel‐free photocatalytic degradation efficiencies of methyl orange (boosted by more than 40%) and picric acid (improved by about 15%).

Different from the above light‐driven photocatalyst‐noble metal based Janus micromotors moving toward photocatalyst side, the motion direction may also be reversed to metal side by material composition and structure designs. For instance, Au‐WO_3_@C three‐component Janus motors were prepared in Ren's group, which moved to metal Au coated side through self‐diffusiophoresis (Figure 6d) and improved the photocatalytic degradation efficiency of sodium‐2,6‐dichloroindophenol by about 70% thanks to the introduced fast fluid flow.^[^ ^177^ ^]^ Moreover, Cai and co‐workers presented a visible light powered bismuth oxyiodide (BiOI)‐based Janus micromotor with one hemisphere coated with metal layer (Au, Pt, Al_2_O_3_) propelled by self‐diffusiophoresis mechanism.^[^ ^178^ ^]^ Exposed to light illumination, the photogenerated electrons from BiOI moved to metal layer, causing net negative charges at metal side. At BiOI side, hydrogen ions were formed from the oxidation of water and concentrated on its surface. For balancing electrical charges, a self‐diffusiophoretic fluid flow of water occurred and mass transfer was accelerated. Furthermore, they developed a novel light‐driven two‐in‐one Janus TiO_2_‐Fe micromotor (degrading organic pollutions by photocatalysis and photo‐Fenton processes, as shown in Figure 6e) by self‐diffusiophoresis process, which moved toward metal Pt direction during photocatalysis and thus increased the photocatalytic degradation first‐order rate constant of rhodamine 6G from 9.98 × 10^−2^ to 23.34 × 10^−2^ min^–1^.^[^ ^179^ ^]^

In addition to the intensively studied Janus structures, helical structure existed widely in nature may also endow materials with outstanding properties, which has been incorporated into photocatalyst‐based motors for mass‐transfer facilitated photocatalysis by Wang and co‐workers.^[^ ^180^ ^]^ Specifically, helix carbon nanocoil/TiO_2_ (CNC/TiO_2_) nanomotors were constructed in their work, and in addition to the self‐diffusiophoresis mechanism‐imparted driving force stemmed from the asymmetric redox reactions (Figure 6f), torque and viscous drag were formed over CNT/TiO_2_ motors in the reactive solution. As a consequence, multimotion modes, including translation, rotating and stirring, were generated ascribed to the unique spiral structure‐imparted various stress situations of CNT/TiO_2_ motors. Significantly, compared with stationary situation, the degradation efficiency of contaminant phenol was increased by 37% thanks to the self‐propulsion motor‐promoted mass transfer rate in multiple dimensions.

**Figure 7:**
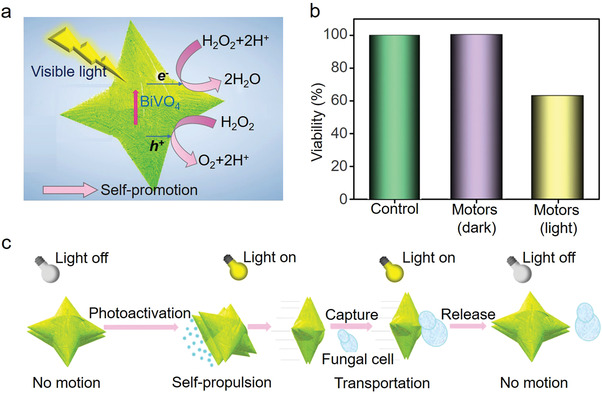
a) Schematic diagram of the photocatalytic activation and the corresponding self‐propulsion mechanism, b) the viability of yeast cells upon the treatment of BiVO_4_ samples for 2 h under visible light illumination, and c) the schematic illustration of visible‐light activated BiVO_4_ micromotors for fungal cell transportation (capture and release). Reproduced with permission.^[^ ^181^ ^]^ Copyright 2019, American Chemical Society.

Moreover, Pumera and co‐workers developed single‐component star‐shaped BiVO_4_ micromotors which showed various trajectories (swimming individually or aggregating with other nearby motors) under visible light illumination (**Figure** **7**).^[^ ^181^ ^]^ In detail, the photoexcited electrons of BiVO_4_ reacted with oxygen dissolved in solution media or H_2_O_2_ molecules for generating reactive oxygen species, and photoexcited holes oxidized water molecules into hydroxyl radicals or oxidize H_2_O_2_ into oxygen and protons, as depicted in Figure 7a. Consequently, an asymmetrical generation of chemical ions was formed on the multifaceted BiVO_4_ micromotors due to their asymmetrical shape and heterogeneous surface with specific crystal planes and different atomic terminations. Thanks to the different diffusion rates over the photoexcited ions, a local electric field and a chemical pressure were formed around the motor, which propelled the motors move forward via self‐diffusiophoretic mechanism. Significantly, these micromotors possessed an amazing autonomous ability to adhere to living yeast cells, disinfect and transport them with controllable manners under light irradiation, as a result, the viability of yeast cells was decreased to 60% in the presence of BiVO_4_ micromotors (Figure 7b,c). This work demonstrated that this kind of visible‐light‐enabled micromotors can function as disinfection motile tools for remediating microbial contamination in water with boosted photocatalytic activity attributed to the coupling effect of self‐propelled mass transfer and high‐efficient reactive oxygen species generated during photocatalysis. Additional, an obvious self‐diffusiophoretic motion was recorded in a co‐flow microchannel over plain catalytic particles TiO_2_ driven by the macroscopic concentration field generated by themselves, and photocatalyst particle moved toward higher reactant concentration due to osmotic pressure differences and diffusion potentials in the case of charges species.^[^ ^182^ ^]^ All these works stressed that self‐diffusiophoretic motion can be achieved over photocatalytic particles that generate concentration gradients originated from their inhomogeneous distribution and the self‐propelled photocatalytic particles do not need to be Janus type.

##### Plasmonic Resonance Effect‐Enhanced Self‐Propulsion

**Figure 8:**
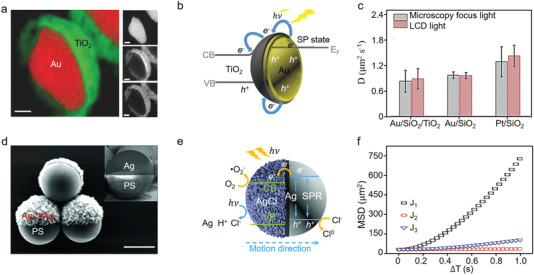
a) Color‐coded elemental map of Au/TiO_2_ nanocapped motor (the scale bar is 50 nm), b) schematic cartoon for the surface plasmon resonance (SPR) effect of Au/TiO_2_ under visible light, and c) average diffusion coefficient values of Au/SiO_2_/TiO_2_, Au/SiO_2_, Pt/TiO_2_ nanocaps with focus light (1 mW cm^–2^) and LCD light (100 mW cm^–2^). Reproduced with permission.^[^ ^183^ ^]^ Copyright 2018, Wiley‐VCH. d) Scanning electron microscopy (SEM) images of as prepared PS/Ag capped particles (inset) and PS/Ag/AgCl Janus particle (the scale bar is 2 µm), e) schematic diagram of visible light absorption process of Janus PS/Ag/AgCl micromotors based on the SPR effect, and f) MSD curves of different Janus particle assemblies (J_1_, J_2_ and J_3_, stand for 1, 2, and a big cluster of Janus particles) in Rhodamine B solution (100 × 10^–3^ mol L^–1^) under blue light irradiation. Reproduced with permission.^[^ ^184^ ^]^ Copyright 2018, Wiley‐VCH.

In addition to inherent self‐propulsion mechanism of motors, surface plasmonic resonance effect of noble metal can also manipulate mass transfer kinetic characters thanks to the promoted electron transport between metal and photocatalysts. For instance, Sánchez and co‐authors developed a nanocap shaped Au/TiO_2_ nanomotor with the diameter of 175 nm (**Figure** **8**a), which displayed facilitated Brownian motion under the excitation of broad‐spectrum visible light.^[^ ^183^ ^]^ As Figure 8b showed, in the presence of liquid crystal display lamp (with an intensity of 100 mW cm^–2^) illumination, surface plasmon resonance effect of Au led to the appearance of nanocap as bright spots. Moreover, more charges being in oscillation would be generated on Au surface upon higher light intensity, which would cause more pronounced plasmon‐imparted photocatalytic effect in turn. Therefore, a boosted proton gradient and fluid shear velocity would be introduced under higher light intensity due to the higher charge separation‐enabled improved fluid flow around the nanocap particles, leading to enhanced Brown motion as well as mass transfer behavior of Au/TiO_2_. Notably, there were two probably factors which may affect Brown motion of Au/TiO_2_ nanocaps during photocatalysis. The first one was the electron diffusion between TiO_2_ and Au (ascribed to the electrons of Au layer got excited upon visible light irradiation), and the second one was the dissipation of a part of incident light energy in form of heat and its diffusion into surrounding fluid solution. To figure out the main reason, control experiments of Pt/TiO_2_ were conducted, and the results demonstrated that the remained electrons of Au transferred to the conduction band of TiO_2_ and TiO_2_ surface thanks to the charge separation, creating an electric field around the nanocap. On the contrary, the protons originated from the oxidation of water at Au layer moved to TiO_2_ and were reduced by the diffused electrons of TiO_2_ layer. As a consequence, a solute fluid flow from Au inner surface toward TiO_2_ outer surface around Au/TiO_2_ nanocaps was formed, which provided a driving force to propel the nanocaps move forward. As for the second factor, Au/SiO_2_, Au/amorphous TiO_2_ and Au/SiO_2_/TiO_2_ nanocaps were constructed as control samples, and the results implied that the thermophoresis (the fast heat transfer away from water‐Au interface to surroundings aided the nanocap movement) was not a significant effect for enhancing Brown motion (Figure 8c). This work proposed a mechanism named plasmonic photocatalytic effect in photocatalytic field based on self‐diffusiophoresis originated from surface plasmon resonance effect, providing a genetic strategy to improve photocatalytic performance by facilitating mass transfer.

Based on the above‐mentioned mechanism, Makarov's and co‐authors constructed visible light‐driven plasmonic Ag/AgCl‐based spherical Janus micromotors PS@Ag/AgCl composed of 2 µm polystyrene (PS) sphere capped with 60 nm thin film of Ag/AgCl, as displayed in Figure 8d.^[^ ^184^ ^]^ As Figure 8e showed, an asymmetric internal electric field was formed around the Janus micromotor due to the different diffusion speeds of Cl^–^ and H^+^ ions induced by the photodecomposition process of AgCl (4AgCl + 2H_2_O → 4Ag + 4H^+^ + 4Cl^−^ + O · ^−^), as a result, the micromotors moved toward the nanocapped PS surface. Importantly, thanks to the couple effect between plasmonic light absorption and photochemical decomposition of AgCl, the kinetic characters of constructed micromotors were promoted in the case of single motors, further demonstrating their motion mechanism of self‐diffusiophoresis. Furthermore, the dynamic characteristics of prepared motors in rhodamine B solution corroborated their self‐propulsion performance (Figure 8f) and promoted mass transfer activity for the future potential applications in environmental remediation.

The previous studies disclosed that light‐driven photocatalyst micro/nanomotors can be constructed with multicomponent Janus nanoparticles consisted of photocatalysts and noble metal (e.g., Au, Pt, Ag, and so on) or photothermal substance (e.g., carbon sphere/tube and carbon bottle), and other special structures such as helix and star‐like heterogeneous photocatalysts. The basic principle of the self‐propulsion mechanism (e.g., self‐thermophoresis and self‐diffusiophoresis) of these micro/nanomotors was the asymmetrical distribution of heat, ions, protons, or photoexcited carriers around motors and their nearby surroundings. Therefore, the formation of heat, ions, protons and photoexcited carriers concentration differences around photocatalyst motors is the key for achieving self‐propelled mass transfer and promoted photocatalytic performance. In this regard, tailored and ingenious material composition and structure designs are welcomed. Moreover, surface plasmonic resonance effect can couple with self‐propulsion mechanism of photocatalyst motors to further enhance mass transfer kinetics, which is worthy to be considered when constructing photocatalysts.

#### 3.1.2 Bubble‐Driven Photocatalytic Motor‐Promoted Mass Transport

**Figure 9:**
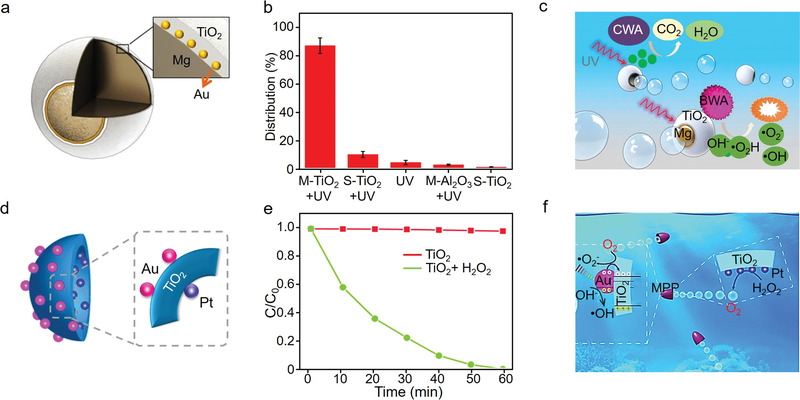
a) Schematic of designed TiO_2_/Au/Mg motor structure, b) spore destruction efficiency under different treatments (“M” and “S” stand for “moving” and “static”, respectively), and c) **s**chematic diagram of the self‐propulsion and photocatalytic degradation of biological warfare agents (BWA) and chemical warfare agents (CWA) by TiO_2_/Au/Mg micromotors. Reproduced with permission.^[^ ^186^ ^]^ Copyright 2014, American Chemical Society. d) Schematic diagram of prepared Pt/TiO_2_/Au semishell micromotor, e) photocatalytic degradation rates of rhodamine B by 0.5 mg mL^−1^ prepared MPP with 5% H_2_O_2_ under solar light irradiation, and f) schematic diagram outlining the solar photocatalytic degradation of organic pollutants with H_2_O_2_ as the fuel. Reproduced with permission.^[^ ^187^ ^]^ Copyright 2016, Royal Society of Chemistry.

Bubble‐driven photocatalyst motors are mainly composed of photocatalysts and reactive metals. These active metal substances can intensively react with water or fuels during photocatalytic reactions and generate lots of air bubbles (predominately include hydrogen and oxygen) to propel the motion of powder photocatalysts.^[^ ^185^ ^]^ Consequently, the mass transfer is accelerated and photocatalytic activity is thereby promoted. For example, Wang's group reported a magnesium‐based Janus micromotor TiO_2_/Au/Mg (Mg served as core, and Au nanoparticles as well as TiO_2_ functioned as shell layers), which generated massive hydrogen bubbles from the positive reaction between water and Mg core, introducing plenty of highly oxidative species from TiO_2_ surface (**Figure** **9**a).^[^ ^186^ ^]^ As illustrated in Figure 9b, the destruction rate of bacillus globigii spores was increased from about 10% with static TiO_2_ to 85% with moving TiO_2_. It was attributed to that these produced hydrogen bubbles propelled the motors to move fast in fluid medium, and thus remarkably facilitated fluid transport and the dispersion of photogenerated reactive species, leading to a significantly improved photocatalytic activity by about 75% (Figure 9c). Similarly, Qu constructed a Pt/TiO_2_/Au semishell motor photocatalyst system, in which Au and Pt nanoparticles were deposited and embedded on the outer and inner surface of TiO_2_, respectively, as displayed in Figure 9d.^[^ ^187^ ^]^ Upon light illumination, the degradation efficiency of rhodamine B over Pt/TiO_2_/Au in the presence of 5% H_2_O_2_ fuel was about 95% higher that obtained over pristine TiO_2_ in 60 min (Figure 9e). It was originated from that the massive O_2_ bubbles generated by the reaction between Pt nanoparticles and H_2_O_2_ imparted the self‐motion of photocatalyst motors and thus accelerated their mass transfer kinetics (Figure 9f), which also increased the dissolved oxygen concentration stemmed from H_2_O_2_ decomposition and hydrogen peroxidase mimetic activity of Au nanoparticles.

**Figure 10:**
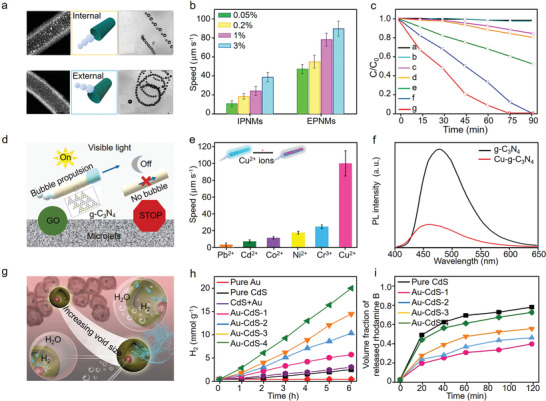
a) Schematic diagram of designed bubble‐propelled internal (IPNMs) and external (EPNMs) propelled nanomotors, b) transfer speeds of motors with different H_2_O_2_ concentrations, and c) photocatalytic degradation rates of rhodamine B (RhB) under different conditions. Letter a to g stands for EPNMs in RhB in the dark, EPNMs in RhB and 0.2% H_2_O_2_ in the dark, RhB solution under light illumination, TiO_2_ nanotubes in RhB under light illumination, RhB plus 0.2% H_2_O_2_ under light illumination, IPNMs in RhB plus 0.2% H_2_O_2_ under light illumination, and EPNMs in RB plus 0.2% H_2_O_2_ under light illumination, respectively. Reproduced with permission.^[^ ^188^ ^]^ Copyright 2017, American Chemical Society. d) Illustration of g‐C_3_N_4_ motors facilitated photocatalytic heavy metal ions removal performance via bubble‐propulsion mechanism, e) detected speeds of g‐C_3_N_4_ micromotors in different heavy metal ion (15 ppm) solutions with 0.25 wt% surfactant and 5 wt% H_2_O_2_, and f) photoluminescence (PL) overlapped spectra of g‐C_3_N_4_ micromotors before and after Cu adsorption. Reproduced with permission.^[^ ^189^ ^]^ Copyright 2018, American Chemical Society. g) Diagram of constructed CdS‐based motor, h) hydrogen production efficiency in the presence of CdS‐based nanostructures (with the void sizes of 40.2, 47.3, 52.1, and 59.7 nm were named as Au‐CdS‐1, Au‐CdS‐2, Au‐CdS‐3, Au‐CdS‐4, respectively) and i) photocatalytic releasing activity of rhodamine B under visible light irradiation. Reproduced with permission.^[^ ^190^ ^]^ Copyright 2019, Elsevier.

Except spherical and semishell Janus structures, Wang and co‐workers developed an internally/externally oxygen bubble‐propelled photocatalytic tubular nanomotor Pt/TiO_2_/Pt (Pt nanoparticles deposited inside and outside of TiO_2_ nanotube), in which structure Pt could react with H_2_O_2_ to generate O_2_ bubbles vigorously (**Figure** **10**a).^[^ ^188^ ^]^ As Figure 10b displayed, the observed increased speeds of internal (IPNMs, about 40 µm s^–1^) and external (EPNMs, about 90 µm s^–1^) propelled nanomotors with the increases in H_2_O_2_ concentrations (from 0% to 3%) demonstrated that EPNMs had greater potential in promoting the mass transfer of Pt/TiO_2_/Pt during photocatalysis in the presence of H_2_O_2_ fuel. Importantly, the photocatalytic rhodamine B decomposition rate over EPNMs was about 30% higher that over IPNMs in 45 min due to the rapid growth and detachment of O_2_ bubbles upon motors (Figure 10c), which promoted the mass transfer behavior of active species during photocatalysis.

Except dye pollution decomposition, bubble‐propelled micro/nanomotors can also be applied in heavy metal ions removal and water splitting for hydrogen generation. For example, Pumera and co‐authors constructed a metal‐free visible‐light photoactivated g‐C_3_N_4_ bubble‐propelled tubular micromotors which showed self‐propulsion property under a variety of heavy metal ion (including Pb^2+^, Cd^2+^, Co^2+^, Ni^2+^, Cr^3+^, and Cu^2+^) solutions, and a highest transfer speed of about 90 µm s^–1^ was obtained over Cu^2+^ solutions (Figure 10d,e).^[^ ^189^ ^]^ Notably, the photoluminescent spectra of g‐C_3_N_4_ tubular micromotors before and after the adsorption of Cu^2+^ unveiled their robust ability in removing heavy metal ions for decontaminating wastewater (Figure 10f). Moreover, Zhang and Hsu constructed self‐propelled yolk‐shell nanostructures with porous shells and mobile cores for enhancing photocatalytic hydrogen generation performance, in which structure Au nanoparticle with the size of about 15.3 nm and CdS with the thickness of about 11.5 nm served as the core and shell, respectively, providing a continuous driving force for mass transfer during photocatalytic hydrogen evolution process (Figure 10 g).^[^ ^190^ ^]^ Compared with pure CdS, the hydrogen evolution efficiency of Au‐CdS‐4 with larger void size was improved by about 8.4 times from 0.36 to 3.39 mmol g^–1^ h^–1^ (Figure 10 h). Moreover, to investigate the mass transport kinetics during photocatalysis, the authors proposed a novel experimental characterization method based on monitoring the mass transport of organic dye molecules (functioning as optical probes) through porous CdS shell for simulating chemical transport and quantitatively comparing mass diffusion kinetics (Figure 10i), and the results disclosed the critical role of void size in modulating mass transfer kinetics and thus boosting photocatalytic hydrogen evolution performance. Notably, ultrafast transient absorption, finite‐difference time‐domain simulation accompanying with the above‐mentioned optical probe characterization substantiated that the improved mass transfer can couple with surface plasmonic resonance effect as well as promoted charge separation rate to further enhance photocatalysis.

These investigations unveiled that bubble‐driven photocatalyst motors facilitate mass transfer by oxygen or hydrogen bubbles during photocatalysis, which are generated from the reaction between active metal and H_2_O_2_ fuel or water. In this regard, the asymmetric deposition or growth of active metal substance on the inner or outer surface of photocatalysts is of great significance for realizing promoted mass transport rate and boosted photocatalytic activity, e.g., pollution degradation, bacteria disinfection, and hydrogen evolution efficiency. More importantly, mass transfer can couple with other impact factors (such as photoexcited carrier migration and separation, and light absorption) to further improve photocatalytic performance.

### 3.2 Artificial Cilia‐Facilitated Mass Transfer

**Figure 11:**
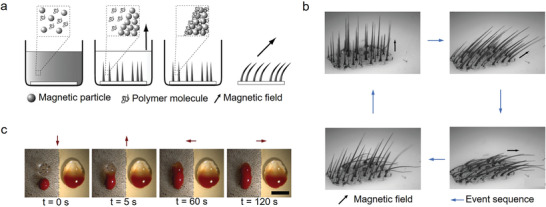
a) Schematic diagram of preparation process of magnetic cilia mimics via template free approach, b) the motion behavior of obtained cilia under magnetic field, and c) fluid mixing ability demonstration of the cilia mimics (two drops of glycerol by periodic actuation of the magnetic cilia mimics (left) compared to diffusive mixing (right) at different time intervals). Reproduced with permission.^[^ ^201^ ^]^ Copyright 2010, American Chemical Society.

Hair‐like structural cilium is one kind of cell organelles, which ubiquitously distributes on the surface of various biological cells in nature and help cells in sensing outside environment and actuate the motion.^[^ ^191^ ^]^ Inspired by natural cilia, many kinds of artificial cilia‐like structures have been developed, which can be propelled by a variety of stimuli, including light, heat, and magnet for the applications in fluid transport, touch and vibration sensing, monitoring, actuators, self‐cleaning devices, and so forth.^[^ ^192^ ^–^ ^199^ ^]^ In this context, R. Superfine and co‐authors prepared high‐aspect‐ratio cilia nanostructures with template magnetically actuated composite polymer rod arrays composed of organic substrate polydimethylsiloxane (PDMS) and magnetic Fe_2_O_3_.^[^ ^200^ ^]^ The attained cilia with the diameter of 200 nm to 1 µm and length of 10 to 25 µm were very flexible, which could induce many kinds of beat pattern under magnet moving underneath the cilia arrays. Ras and co‐workers optimized the preparation process and structure of artificial cilia via a simple template free approach (**Figure** **11**a), and characterized the response as well as actuation role of the constructed cilia structure for mixing liquid drops under magnetic field (Figure 11b,c).^[^ ^201^ ^]^ It indicated that with cilia mimics, two drops could be mixed together in shorter time (120 s), demonstrating the important promotion effect of artificial cilia on fluid flow as well as mass transfer. Moreover, Onck and co‐workers studied the impact of artificial cilia on microfluidic propulsion through comprehensive analyzing experiment results and simulations, further verifying the vital role of artificial cilia structure in facilitating fluid flow for efficient mass transfer.^[^ ^202^ ^]^

**Figure 12:**
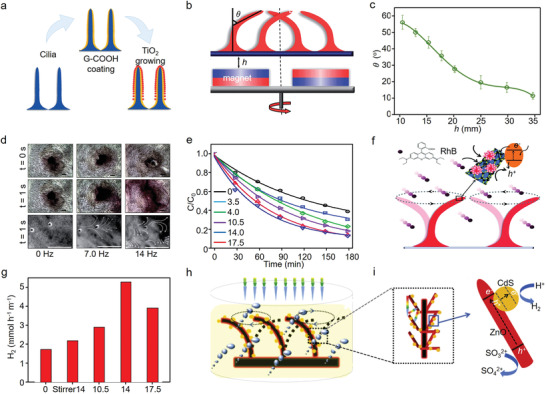
a) Schematic diagram of the structure of prepared photocatalytic artificial cilia, b) magnetically actuated artificial cilia and c) the corresponding title angles changed with the distance between cilia and magnet, d) visualization of the fluidic mixing performance and the corresponding net fluid flow in an aqueous solution for the photocatalytic cilia at various actuation frequencies, e) photocatalytic degradation efficiency of rhodamine B under different frequencies of magnetic actuation, and f) schematic illustration of structure and ciliary motion for interior mass transfer of the inner‐motile photocatalyst film for the photocatalytic degradation of rhodamine B (RhB). Reproduced with permission.^[^ ^203^ ^]^ Copyright 2014, Royal Society of Chemistry. g) Photocatalytic hydrogen evolution rates of prepared ZCS‐B and h) schematic illustration of the mass transfer as well as i) charge transfer in the inner‐motile ZCS‐B film for hydrogen evolution. Reproduced with permission.^[^ ^205^ ^]^ Copyright 2014, Elsevier.

On the basis of the above‐mentioned theoretical and experimental results, plenty of research works focusing on artificial cilia‐constructed photocatalytic materials and systems were carried out, and some typical representatives were summarized in **Table** **2**. For example, Lu and co‐authors constructed a new type of inner‐motile photocatalyst films with artificial cilia‐like structure via template free method, which were self‐supporting and could be bent to any specific angles ranged from 10° to 55° upon the stimulus of magnetic field, as depicted in **Figure** **12**a–c.^[^ ^203^ ^]^ With the impactful and flexible cilia‐like structures, the net vortex could be observed around the cilium, and the flow vortexes were more obvious when actuated at higher frequency of 14 Hz, shown in Figure 12d. These vortexes changed randomly with time and space of flow velocities and directions, leading to irregular fluid motions which would quickly disperse and homogenize the reactants. As a result, the photocatalytic degradation rate of rhodamine B over prepared TiO_2_‐based cilia array was improved by about 30% (summarized in Table 2) due to the boosted interior mass transfer and accelerated desorption of the degradation reactive species (Figure 12e,f). Moreover, the photocatalytic degradation activity of constructed photocatalytic cilia was almost not changed after five runs for 15 h, showing the excellent recycling stability for practical application. In addition, Tseng et al. corroborated that compared with photocatalyst film, cilia‐like structure endowed photocatalyst with higher surface area, better light harvesting ability and greater practical durability, which would further enhance the photocatalytic activity of artificial cilia photocatalysts.^[^ ^204^ ^]^

**Table 2:** Summary of some typical representatives of artificial cilia‐constructed photocatalytic materials and their applications

Cilia composition	Cilia structure	Catalytic applications	Enhancement	Enhancement mechanism	Ref.
PDMS/TiO_2_	TiO_2_ particles deposited on PDMS array	Rhodamine B degradation	30%	Improved mass transfer	[203]
Cu/*it*PI	containing Cu, Cu_2_O and polyimide	CO_2_ reduction	2 times		[204]
PDMS/ZnO@CdS	CdS quantum dots grow on ZnO nanorods deposited on PDMS array	H_2_ evolution	2.7 times	Promoted mass transfer, light absorption, hydrogen desorption, carrier separation	[205]
PDMS/ZnO/BiVO_4_	BiVO_4_ nanoparticles grow on ZnO nanosheets deposited on PDMS array	Rhodamine B degradation	20%	Boosted mass transfer, light absorption, charge separation	[206]
PDMS/ZnO@TiO_2_	TiO_2_ nanoparticles grow on ZnO nanorod deposited on PDMS array	Rhodamine B degradation	35%	Enhanced mass transfer, product desorption	[207]
PDMS/RGO/TiO_2_	TiO_2_ nanosheets grow on RGO deposited on PDMS	Rhodamine B degradation	Three folds	Increased mass transfer, light absorption, charge separation	[208]
SnFe_2_O_4_	SnFe_2_O_4_ nanorod arrays grow on PDMS film	Rhodamine B degradation	23%	Accelerated mass transfer	[125]
PDMS/ZnO	ZnO nanorod arrays grow on PDMS film	Methylene blue degradation	40%		[209]

To further take advantages of cilia‐like structure, Lu's group constructed ZnO nanorods on the surface of cilia and deposited CdS quantum dots on ZnO branches, forming heterostructure magnetically actuated artificial cilia film (ZCB‐S), which exhibited 2.7 times higher photocatalytic hydrogen evolution activity (up to about 5500 µmol h^–1^ m^–1^ under magnet‐actuation with 14 Hz) that without magnetic actuation situation (Figure 12 g).^[^ ^205^ ^]^ The obvious improvement in photocatalytic performance was ascribed to the natural manipulation ability of cilia in fluid actuators, which promoted mass transfer and the desorption of hydrogen from active sites, facilitating continuous photocatalytic hydrogen generation reaction (Figure 12 h). It was worth noting that the improved carrier separation rate stemmed from the heterojunction structure between CdS and ZnO also made contribution to the enhanced photocatalytic hydrogen evolution efficiency. This work opened an access to deal with the stubborn problem of hydrogen desorption during photocatalytic water splitting reaction for boosting hydrogen production performance. Similarly, ZnO/BiVO_4_ nanosheet arrays, ZnO@TiO_2_ mushroom arrays and RGO/TiO_2_ nanosheets were exclusively immobilized on magnetically actuated artificial cilia films, and in the presence of ciliary motion, photocatalytic activities were obviously improved ascribed to the boosted inner mass transfer.^[^ ^206^, ^207^, ^208^ ^]^

Additionally, Chen's group developed SnFe_2_O_4_ nanoparticles on the surface of magnetic artificial cilia and studied the impact of cilia rotation mode on photocatalytic degradation efficiency.^[^ ^125^ ^]^ The results signified that 20% higher photocatalytic activity was achieved over SnFe_2_O_4_ cilia photocatalyst compared with that obtained over photocatalyst without cilia structure because of the rapid and uniform inner mass transfer. Moreover, Yeom and co‐workers also studied the effect of mass transfer on photocatalytic performance through constructing ZnO nanowire array cilia on PDMS film substrate, which exhibited great stability to mechanical bending, stretching, contact and temperature variation.^[^ ^209^ ^]^ With magnetic bar stirring to introduce fluid flow during photocatalytic reaction, the photodegradation ability of ZnO cilia was remarkably improved by about 40% (summarized in Table 2), and the corresponding reaction rate constant was increased from 0.0018 (without fluid flow) to 0.0074 min^–1^ (with fluid flow). It was attributed to that the fluid‐flow‐improved mass transfer played a significant role in photocatalytic activity, implying that not only flexible organic polymer‐based cilia but also inorganic cilia structure can efficiently promote mass transfer and thus improve photocatalytic degradation activity as well as photocatalytic hydrogen evolution performance.

## 4 Summary and Outlook

### 4.1 Summary

Past decades have witnessed the fast development of photocatalytic technology and plenty of modification methods have been proposed based on broadening light absorption, promoting carrier separation and migration as well as facilitating surface molecule reactions, which give access to notable achievements. In addition to the above‐mentioned strategies, mass transfer behavior modulation also plays a pivotal role in determining final photocatalytic performance, due to the short‐live active species (e.g., photoexcited electrons and holes, hydroxyl radical, and superoxide radical) and superfast redox process during photocatalysis. Significantly, previous studies implied that efficient mass transfer is beneficial for improving photocatalytic performance, whereas too fast mass transfer rate will lead to poor photocatalytic activity ascribed to the lower residence time of active species on photocatalytic surface. Therefore, it is necessary to optimize kinetic characteristics of mass transfer for boosting photocatalytic efficiency.

In this regard, plenty of photocatalysts with ingenious structure and composition designs have been developed to facilitate mass transfer based on theoretical simulation and calculation. Among them, micro/nanomotor photocatalysts and artificial cilia photocatalysts stand out in terms of promoting photocatalytic hydrogen evolution and organic pollution degradation via modulating mass transfer. Specifically, self‐propulsion behavior can be realized over carbon bottle/photocatalyst without Janus structure under infrared light illumination through self‐thermophoresis effect, which simplified the fabrication process of photocatalytic micro/nanomotors. Additionally, self‐propelled micro/nanomotors with Janus, helical and heterostructures such as TiO_2_‐Au, Au‐WO_3_@C, ZnO/Pt, CNT/TiO_2_, BiVO_4_ and so forth were constructed, which exhibited self‐driven transport behavior and boosted mass transfer rate under infrared light irradiation via self‐diffusiophoresis mechanisms. Except light‐driven motors, some multicomponent Janus‐structured photocatalysts including TiO_2_/Au/Mg, Pt/TiO_2_/Pt semishell and Pt/CNT/Pt nanotube were also developed, in which reactive metal reacted with water or fuel to generate massive bubbles, driving photocatalysts to move rapidly during photocatalysis. It is worth noting that surface plasmonic resonance effect can couple with self‐propulsion effect to further promote mass transfer and photocatalysis. Additionally, photocatalytic artificial cilia which can be actuated by magnetic field were mainly constructed with magnetic particles and organic flexible PDMS. Compared with the above motors, the modulation of mass transfer behavior was more accessible over artificial cilia photocatalysts by controlling the parameter of cilia and magnet rotation speed. Moreover, cilia structure can endow photocatalysts with increased light absorption efficiency and reusability for real applications. All these impactful investigations provided viable and robust routes to manipulate mass transfer kinetics in fluid field, beneficial for enhancing photocatalytic performance and facilitating practical applications of photocatalytic technology. In a wider perspective, it will promote the developments of self‐propulsion system, photonics, molecular dynamics, magnetics and other interdisciplines.

### 4.2 Future Outlook

**Figure 13:**
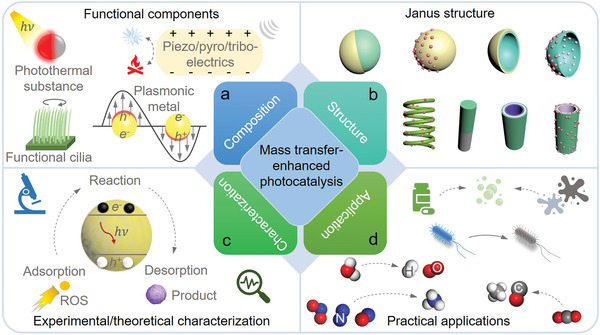
Schematic diagram of the future development of mass transfer‐boosted photocatalytic performance.

Although great progresses have been made, there is still much room to enrich the existing research for the future development of photocatalytic technology in terms of mass transfer modulation, as illustrated in **Figure** **13**. First of all, the number and types of micro/nanomotors explored for facilitating mass transfer are very limited at present, which could not present sufficient theoretical and experimental guidance for those working in this field. For artificial cilia, besides magnet‐actuation mechanism, other robust actuation methods should also be considered and applied to stimulate the self‐propulsion of artificial cilia photocatalyst systems by incorporating light, heat, electricity, or acoustic field responsive substances into photocatalytic cilia structure, realizing multimode self‐propulsion of photocatalyst cilia. Moreover, viable materials with piezoelectric, pyroelectric, and triboelectric properties are expected to combine with cilia photocatalysts, in which piezo‐/pyro‐/tribo‐electric field can be generated under deformation‐enabled strain/thermal fluctuation/friction for promoting the separation of photoexcited carriers (Figure 13a). Thus, in addition to boost mass transfer, reasonable cilia structure and photocatalyst composition design will lead to promoted carrier separation and efficient light absorption. As a result, multiform energy sources can be efficiently utilized for achieving synergistically enhanced photocatalytic performance throughout almost the whole photocatalytic process.

Second, as illustrated in Figure 13b, more micro/nanomotors with tailored structures (e.g., Janus nanoparticle/tube/rod/fiber, core‐shell structure, core‐shell‐shell structure, yolk‐shell, helical structure and other asymmetric structures) and functional compositions (including plasmonic resonance metal, active metal and photothermal substance to endow the motors with certain superpower to harvest surrounding low‐density energy and convert them into their own transport kinetics, Figure 13a) are highly desired to accelerate mass transfer efficiency during photocatalysis for boosting photocatalytic activity. It is worth figuring out that fuel‐free micro/nanomotors need to be considered and predominantly developed due to the serious energy shortage. Notably, for bubble‐facilitated mass transfer, the influence of increased dissolved oxygen and hydroxyl radical concentrations stemmed from the reaction between active metal and water or fuel solution on photocatalytic performance should also be taken into account and analyzed, which also plays a critical role in final photocatalytic performance.

Third, as mentioned in the manuscript, there is an optimum value of mass transfer for photocatalysis, and therefore a universal design to determine the optimum mass transfer rate is necessitated for the development of high‐performance photocatalytic systems and their practical applications. In this regard, advanced characterization techniques for recording dynamic transfer behavior and redox reaction process of reactive species during photocatalysis and theoretical simulation/calculation methodologies (Figure 13c) need to be explored to optimize mass transfer parameters for high‐efficient photocatalysis. On this basis, the mechanism how mass transfer behavior modulates photocatalytic activity should be investigated thoroughly and systematically as well, which will provide critical and comprehensive guidelines for researchers who are interested in this field and other related disciplines.

Finally, applying photocatalytic technology into practical applications, including efficient hydrogen production, CO_2_ reduction into CO, CH_3_OH and other fuels, NO*_x_* conversion, antibiotics and organic pollution degradation, virus and bacteria disinfection, and so on (Figure 13d), is of great social and economic significance for alleviating serious energy and environmental problems. Despite great efforts have been made, converting photocatalytic technology into real applications is still a challenge and one of the main reasons is the low energy efficiency. From this point of view, it will be a long journey for us to achieve tremendous improvement in promoting photocatalytic efficiency and practical application via routine modification methods because the stability, cost, preparation process, energy conversion efficiency and reusability of photocatalysts need to be considered during industrialization. Therefore, innovations in techniques and science centering on semiconductor materials, efficient energy capture/conversion/storage/utilization and other related fields are expected to accelerate the industrialization and practical application processes of photocatalytic technology.

## Conflict of Interest

The authors declare no conflict of interest.
